# Design of high-performance parallelized gene predictors in MATLAB

**DOI:** 10.1186/1756-0500-5-183

**Published:** 2012-04-10

**Authors:** Sylvain Robert Rivard, Jean-Gabriel Mailloux, Rachid Beguenane, Hung Tien Bui

**Affiliations:** 1 Département des sciences appliquées, Université du Québec à Chicoutimi, 555 blvd de l’Université, Chicoutimi, QC, G7H 2B1, Canada; 2 Royal Military College of Canada, Kingston, ON, K7K 7B4, Canada

## Abstract

**Background:**

This paper proposes a method of implementing parallel gene prediction algorithms in MATLAB. The proposed designs are based on either Goertzel’s algorithm or on FFTs and have been implemented using varying amounts of parallelism on a central processing unit (CPU) and on a graphics processing unit (GPU).

**Findings:**

Results show that an implementation using a straightforward approach can require over 4.5 h to process 15 million base pairs (bps) whereas a properly designed one could perform the same task in less than five minutes. In the best case, a GPU implementation can yield these results in 57 s.

**Conclusions:**

The present work shows how parallelism can be used in MATLAB for gene prediction in very large DNA sequences to produce results that are over 270 times faster than a conventional approach. This is significant as MATLAB is typically overlooked due to its apparent slow processing time even though it offers a convenient environment for bioinformatics. From a practical standpoint, this work proposes two strategies for accelerating genome data processing which rely on different parallelization mechanisms. Using a CPU, the work shows that direct access to the MEX function increases execution speed and that the PARFOR construct should be used in order to take full advantage of the parallelizable Goertzel implementation. When the target is a GPU, the work shows that data needs to be segmented into manageable sizes within the GFOR construct before processing in order to minimize execution time.

## Findings

### Background

Since the early beginnings of the human genome project, numerous research groups have developed computerized approaches to studying human genetics [1,2]. In order to deal with the high volume of accumulated biological data, geneticists and molecular biologists require faster algorithms. This is important as the amount of data in gene research roughly doubles every six months whereas computer processing speeds improve at a much lower rate. Thus, speed increases due to hardware improvement alone cannot keep up with the amount of data to process, and therefore, optimization of the existing tools is required.

In bioinformatics, one of the main interests is studying deoxyribonucleic acid (DNA) sequences, which are of fundamental importance in understanding all living species. DNA molecules are at the base of hereditary information and are composed of four types of nucleotides: adenine (A), thymine (T), guanine (G) and cytosine (C). Certain segments of the DNA strand contain the necessary information for protein synthesis and these are called genes. Genes include two sub-sections called coding regions (exons) and non-coding regions (introns). As of the time of publication, it is estimated that several thousands of all human genes have yet to be discovered [3].

In general, bioinformatics applications need to process massive quantities of data.

In the near future, it is the authors’ belief that certain applications will require the processing of a large number of complete genomes. This can occur, for instance, in the case of comparative analyses of individual genomes over an entire population. To facilitate this process and to encourage geneticists to use this promising computerized venue, it is necessary to develop tools with the following characteristics [4]:

· Easy to install locally;

· ability to train and test the programs independently;

· availability of the source code;

· fast processing speeds;

· freedom from excessive licensing restrictions.

The process of discovering genes has traditionally been done in genetics laboratories and is often seen as being long and expensive. Since the beginning of the 21^st^ century, a digitized version of the complete human genome has been made publicly-accessible. It has since been speculated that part of gene discovery could be done digitally using computer algorithms. This task is called gene prediction and involves analyzing a DNA sequence and identifying regions that code for protein. While gene prediction is of great interest, the amount of data that needs to be processed is very large at around 6 billion bps and the required mathematical operations are time consuming. It is therefore important to find techniques that are accessible to geneticists that can process very large amounts of data within a short time frame.

Over the last three decades, digital signal processing (DSP) techniques have been developed for the identification of protein coding regions, both in humans and in others species [5,6]. Fickett [7] was the first to propose the use of an autocorrelation function (ACF) to identify the periodicity within the exons of a genomic sequence. Over the years, other methods have been proposed including frequency analysis using the Fast Fourier Transform (FFT), the autoregressive model and the hidden Markov model [2,4].

The purpose of this work is to offer geneticists and molecular biologists the tools for the prediction or identification of new genes using existing complementary strategies. The objectives of this research are to render this approach fast, reliable, accurate and easy to use, thus requiring little training. Furthermore our approach can work with several types of genomes such as humans, plants or other various organisms.

This work focuses on predicting genes using frequency analysis with FFTs and with an equivalent technique known as Goertzel’s algorithm [8]. These methods have a proven detection rate that can surpass 80% [9], and the results in [10] further demonstrate the reliability of this method. Specifically, this work’s objective is to develop parallel computing techniques that allow for gene prediction algorithms to run at a higher speed on conventional desktop computers. While there are several existing solutions in the literature [11-13] that yield good results, they are often limited to small sequences of a few thousand bps. We believe that the performances of these previously proposed solutions are not yet adequate since linked markers are often separated by millions of bps [14]. This work specifically targets large sequences and makes sure the processing time remains low in order to apply these techniques in actual day to day genetics research. These results are then validated against a specific gene for which the information is well-known. More importantly, the designs proposed herein are implemented using MATLAB rather than developing custom software and/or hardware solutions such as FPGAs or ASICs. The reason is that gene prediction tools often do not match the researcher’s needs. Tailoring existing software/hardware solutions for a specific requirement often involves modifying the design which demands a certain level of expertise. We have witnessed first-hand how time consuming the process of tapping FPGA power [15] can be in this scenario, but most importantly, how inflexible it becomes when the needs of the application change. Since MATLAB is a multi-platform tool with a relatively simple yet powerful programming language that does not require compilation, the difficulties encountered with a custom software/hardware approach are mitigated.

## Frequency analysis in gene prediction

One of the crucial steps in gene prediction is the identification of coding regions. It has been well-established that these coding regions have repeating nucleotide sequences that exhibit a periodicity of three [16-18]. To detect this periodicity, and thus to identify coding regions, the typical approach is to perform frequency analysis using the Discrete Fourier Transform (DFT). Since the straightforward implementation of the DFT is not computationally efficient, the FFT is often preferred. The FFT has been extensively covered in literature, and its optimization has been thoroughly explored [13,17,19,20]. A gene prediction algorithm based on the FFT can therefore yield good results.

The FFT is an operation that takes *S* samples of a time-domain or a space-domain signal and outputs the amplitude and the phase information for a series of *S* frequencies. While all this information may be required in many applications, only the amplitude of one frequency needs to be considered in gene prediction. This frequency of interest, which corresponds to a periodicity of three in the nucleotide sequence, is equal to a third of the sampling frequency (*f*_*s*_*/3*). The remaining phase and amplitude information provided by the FFT are discarded. Thus, for applications that only require a small number of frequencies, such as gene prediction, the use of the FFT is not optimal. A more efficient approach is to use the Goertzel algorithm which is given by:

(1) $$y\left[n\right]=x\left[n\right]+2\cos(2\pi f_{n})y\left[n-1\right]-y\left[n-2\right]$$

In (1), *x* is the input, *y* is the output and *n* is the sample number. Since gene prediction relies on the detection of a periodicity of three in the sequence, the normalized frequency *f*_*n*_ can be replaced by 1/3. In that case, the coefficient of the *y[n-1]* term becomes −1 and the equation can be rewritten as:

(2) $$y\left[n\right]=x\left[n\right]-y\left[n-1\right]-y\left[n-2\right]$$

In this form, the Goertzel algorithm no longer requires multiplications or fractional terms. This allows for a faster and more efficient implementation. It should be noted, however, that MATLAB uses double precision arithmetic when performing certain functions, including the Goertzel algorithm. While the full benefits of having such a simple algorithm are not always apparent, preliminary tests have shown a 28% increase in speed.

After processing *N* elements using equation (2), the final result is obtained with the following equation, where *P*_*X*_ is the power at frequency *f*_*s*_*/3* of nucleotide *X* :

(3) $$P_{X}=y_{N}^{2}+y_{N}^{2}+y_{N}\cdot y_{N}{}_{-1}$$

This process is done for all values of *X* (A, T, G and C) and the results are added together to provide the final output.

### General implementation

**Figure 1:**
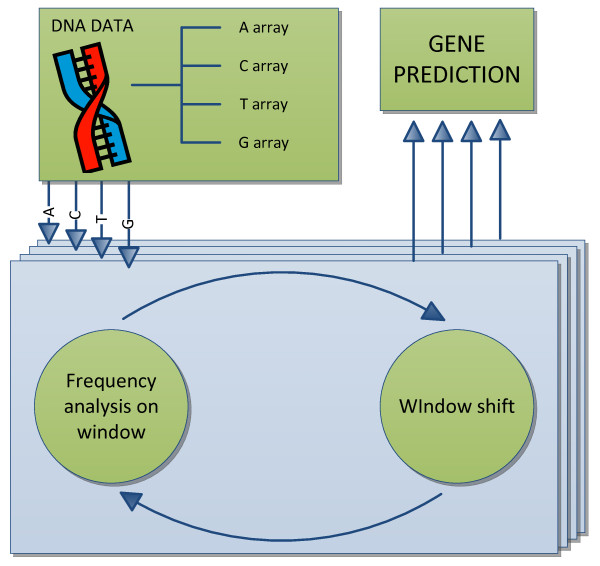
Block diagram of the system.

**Figure 2:**
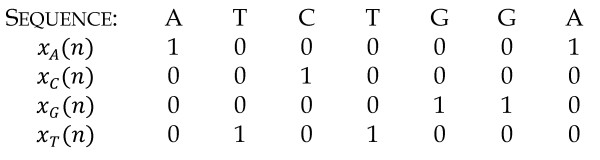
DNA sequence converted into its binary counterparts.

A block diagram of the gene prediction algorithm is shown in Figure 1. It shows that the first step in gene prediction is to separate the DNA sequence into four different vectors containing numerical values. This conversion is what allows for the use of DSP in DNA analysis. While numerous techniques can be used to perform this conversion [21], the one used in this work was proposed by Voss [22]. The approach consists of converting the DNA sequence into four binary vectors, each corresponding to a type of nucleotide. These vectors will contain ‘1’ at a given position if the corresponding nucleotide is of that type. If not, that vector position will be ‘0’. An example of such conversion is shown in Figure 2.

**Figure 3:**
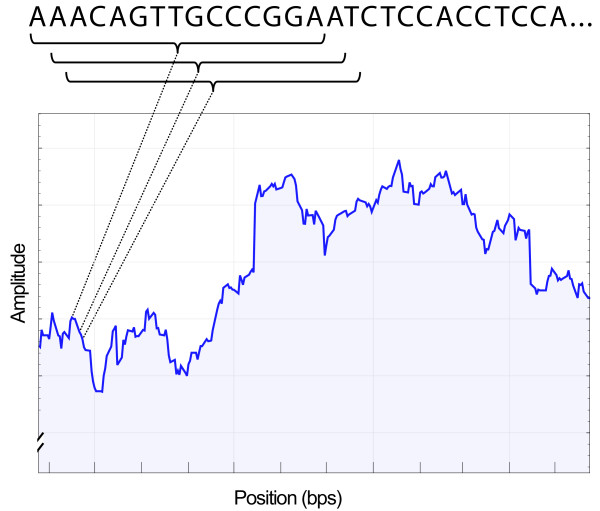
Illustration of the sliding window approach.

In gene prediction, frequency analysis is performed on a small window at a time. Within this window, an FFT or Goertzel’s algorithm is executed in an attempt to detect a periodicity of three. Once this is completed, the window is shifted by one nucleotide before frequency analysis is performed again. This process continues until the whole DNA sequence has been analyzed. At that point, the results from all four vectors are combined to generate the final output. Figure 3 illustrates this process by showing how an output figure is generated. In such a figure, an area with larger amplitudes indicates a higher probability of containing a coding region.

The choice of window size affects the quality of the results. It has been shown that long window sizes remove specificity from the analysis which makes it more difficult to determine the boundary between an intron and an exon. On the other hand, windows that are too small tend to yield noisy results. Mahmood et al. [23] suggest that a size of 351 bps would provide a good balance between noise and specificity; [10] and [19] also demonstrate why 351 is a solid choice.

## Design

There are several ways of implementing frequency analysis in MATLAB. They are categorized according to the nature of the algorithm used to perform the frequency analysis and the processing device used to do so. These are described in the following subsections.

### Single core implementation

In a typical MATLAB working environment, executed instructions will normally run on a single core. In such an environment, gene prediction can be implemented using any of the following implementations.

#### Goertzel

The most straightforward approach to implementing the Goertzel algorithm is to use the built-in function (*goertzel)* provided by MATLAB. The function is stored in an .m file (goertzel.m) which contains validation commands as well as a call to a highly-optimized pre-compiled function called goertzelMEX.

By profiling the goertzel.m function, it was discovered that a large amount of time is spent performing validation commands instead of the Goertzel algorithm itself. In an effort to improve processing time, it is possible to bypass the validation commands and execute the goertzelMEX function directly. To do so, the data sent to the goertzelMEX function need to be formatted properly since the validation process is no longer performed. Given the correct data format, the results produced are the same as with the goertzel.m file while the execution time is significantly reduced.

While the goertzel.m and goertzelMEX functions have been provided with MATLAB, the algorithm can be greatly simplified when used in gene prediction. In such case, it is possible to implement the algorithm directly using equations (2) and (3) which is expected to yield better results.

#### FFT

In addition to the Goertzel algorithm, MATLAB also provides an FFT function (*fft*). Since the hardware implementation and optimization of the FFT have been the topic of many papers, it is expected that a parallelized implementation would yield good results.

### Multi-core implementation

In recent years, it has become common for personal computers to be equipped with multiple cores that can often handle a higher number of threads. In order to make use of these cores, it is possible to instruct MATLAB to use more than one processor when the algorithm is adapted for such parallelism.

In a typical gene prediction algorithm, frequency analysis is sequentially performed on a window of data before moving to the next one. Since each window is independent, frequency analysis can be performed on many windows in parallel, if the resources are available. MATLAB allows for these processes to run in parallel on different threads/cores using the PARFOR construct. PARFOR can be used with any of the previously mentioned implementations.

### GPU implementation

Many modern computers are equipped with graphics cards that contain one or more GPU. Each GPU contains a large number of streaming processors which can be used for parallel processing. While these cards have historically been developed for video processing, they can now be used to accelerate calculations in MATLAB. To access the processing power of these cards within MATLAB, several toolboxes are available including AccelerEyes’ JACKET and the parallel toolbox from MATLAB R2011b. Prior versions of MATLAB provided basic GPU functions, but were too limited to be of interest. For instance, it was not possible to index an array stored in GPU memory. It should be noted that all these technologies call upon NVIDIA’s CUDA technology [25] and are not available on Open Computing langage (OpenCL)-only cards.

#### Matlab R2011b

MATLAB’s parallel toolbox offers several commands that allow for algorithms to be executed on a GPU. Unfortunately, there are no commands that are equivalent to PARFOR that can execute multiple FOR loops in parallel on the GPU. While the command *arrayfun* does offer similar functionality, it removes some of the flexibility needed for this particular algorithm given our need for processing a sliding window within an array. This method was therefore not considered further.

#### JACKET

JACKET is a commercial package that allows MATLAB algorithms to execute parts of their code on GPU. One of the main advantages of using JACKET is the provided GPU counterpart to the PARFOR construct (GFOR). It is therefore a simple task to adapt the MATLAB code to make use of the GPU.

JACKET offers two methods to accelerate the current gene prediction scheme in MATLAB. The first method is to access the FFT function that is implemented on the GPU. The second method, which can be used in conjunction with the first one, is to parallelize the loops using the GFOR command. GFOR, similarly to the PARFOR command, helps to parallelize the operations by distributing them over GPU streaming processors. While this construct does have some limitations (found in JACKET’s documentation), they are not prohibitive for the current algorithm.

## GPU implementation analysis

**Figure 4:**
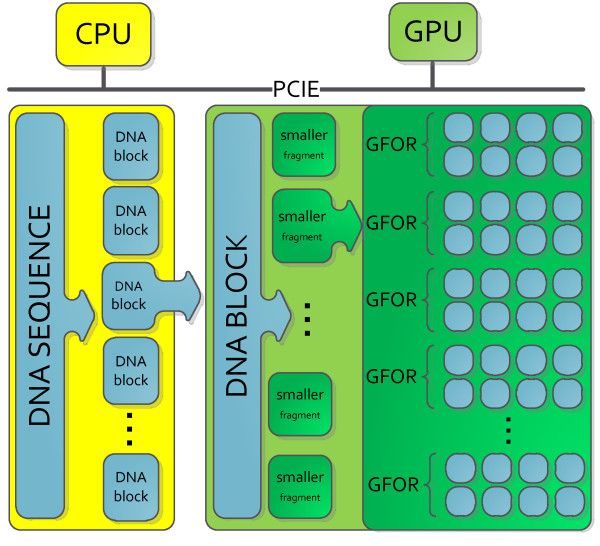
Procedure for breaking down large DNA sequences for processing on GPU.

To make use of the GPU, the chosen DNA sequence must first be split into DNA blocks. These DNA blocks are then sent in sequence to the GPU for processing. When the DNA block is on the GPU, it is broken down further into DNA fragments in order to be processed by separate parallel GFOR instances. This process is illustrated in Figure 4. The difficulty encountered in this implementation revolves around finding the optimal number of GFORs to run in parallel for a given sequence. While a large number of GFORs may improve parallelism, it also consumes the processing resources on the GPU. When the resources are depleted, JACKET’s maintenance algorithm is called to rectify the situation. This process slows down the execution of the algorithm and should be avoided when possible. The goal is therefore to maximize the number of GFORs without depleting the available resources.

Through preliminary tests, it was found that the FFT implementation on the GPU performs much better than any of the GPU Goertzel implementations (Goertzel using the CPU, on the other hand, is fast and is easier to implement). This is expected as FFTs are algorithms that can be parallelized in a straightforward manner whereas the Goertzel algorithm is a recursive one and therefore, does not lend itself easily to parallelization. The GPU implementations will thus only focus on the FFT. Processing times when using Goertzel on a GPU will nonetheless be provided for the purpose of comparison.

A GPU card only has a limited number of parallel processors (*PU*_*TOTAL*_). Knowing that the GPU FFT function requires these resources, the maximum number of GFOR loops (*N*_*GFOR1*_) is given by:

(4) $$N_{GFOR1}\leq \frac{PU_{\mathrm{TOTAL}}}{4\times \left(PU_{\mathrm{FFT}}+PU_{\mathrm{MISC}}\right)}$$

The equation states that four GPU implementations of the FFT need to be present during each GFOR loop to account for each of the nucleotide arrays. The implementation requires *PU*_*FFT*_ processing units for the FFT itself and requires extra processing units (*PU*_*MISC*_) for miscellaneous operations such as the power of two and the summing of the power spectra.

As previously stated, this equation needs to be respected so that the number of processing units does not exceed the available resources. Otherwise, JACKET will reallocate the GPU resources which slows down the execution of the algorithm.

In addition to the processing unit limitation, the implementation also needs to consider the memory constraints. Since every iteration requires a certain amount of GPU memory, the total amount of GPU RAM available also limits the maximum number of GFORs. This constraint can be summarized as follows:

(5) $$N_{GFOR2}\leq \frac{MEM_{\mathrm{TOTAL}}}{4\times \left(MEM_{\mathrm{FFT}}+MEM_{\mathrm{MISC}}\right)+MEM_{\mathrm{SETUP}}}$$

This equation is similar to equation (4) with the exception that it also includes a *MEM*_*SETUP*_ term which accounts for the memory required to manage each different GFOR in order to combine them at the end. The maximum number of GFORs is given by the minimum between *N*_*GFOR1*_ and *N*_*GFOR2*_:

(6) $$N_{\mathrm{GFOR}}=\min\left(N_{GFOR1},N_{GFOR2}\right)$$

In the specific case of the chosen test system, *N*_*GFOR1*_ was smaller than *N*_*GFOR2*_ which means that all parallel resources can be used before the GPU memory is filled. GPU memory is an important issue to consider because of the communication overhead it involves. Sending data to and from the GPU is slow when compared to the GPU processing time. In order to minimize these data transfers, it is efficient to divide the DNA sequence into large blocks before sending them to the GPU, albeit with some caveats which will later be discussed. It is important, however, to not surpass a predefined limit, since larger blocks will produce out of memory errors while the GPU computes. Fortunately, the test GPU used in this work has 1 GB of RAM which is sufficient to store the optimal DNA block size.

**Figure 5:**
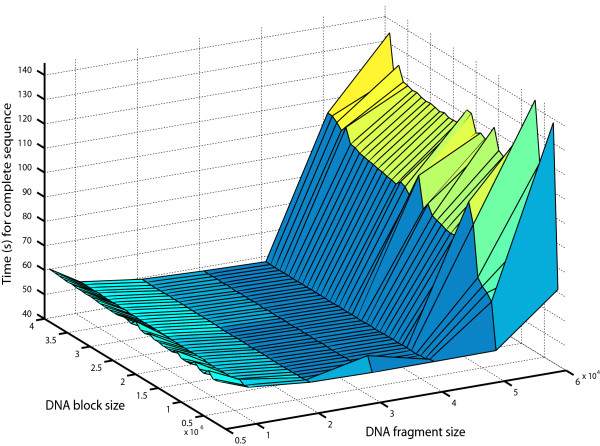
Processing time for a sequence of 15 million bps with varying DNA block size and DNA fragment size.

To determine the optimal DNA fragment size, an extensive series of tests have been performed. DNA blocks of different sizes were sent to the GPU and were processed by implementations using different quantities of GFORs. The results of these tests are shown in Figure 5. It is worth noting that, in this figure, the number of GFORs is not shown explicitly. Instead, the DNA fragment size processed by the GFOR instances is shown. The number of parallel GFORs can be deduced from the fragment size as it is proportional to it. The results shown in Figure 5 also include all data transfers between CPU and GPU memory. In an effort to make the comparisons fair, the processing times have been normalized for a large global sequence of 15 million bps. This is an estimated maximum sequence size over which a gene prediction algorithm needs to be executed in order to find a candidate gene between two genetic markers. The basis for this estimation can be found in [14] where the two markers were separated by 4 centiMorgan (cM) which roughly corresponds to 4 million bps. From Figure 5, it can be determined that using DNA fragment sizes between 20,000 and 40,000 bps yields the lowest processing times. A smaller DNA fragment size would not fully benefit from all the available parallel processing power and therefore, perform poorly. This can be shown in Figure 5, where the processing time increases as the fragment size drops to 5,000 bps.

## Results

To evaluate the processing time of the different gene prediction implementations, tests were performed on DNA segments retrieved from the NCBI library (GRCh37.2). For the purpose of this work, the HFE2 (hemochromatosis type 2 in the human genome) gene [Accession number EMBL:AY372521] was chosen and large DNA segments containing this gene were extracted from the database. The HFE2 gene has four different variants each having a slightly different set of exons. These different exons are presented in Table 1. The human genome used for this test contains all the different exons whose beginning and ending positions are shown in Table 2.

**Table 1:** data on HFE2 gene on chromosome 1

	**Exons**	**Total length of coding region (bps)**
Transcript variant a	1,2,3b,4	2234
Transcript variant b	1,3b,4	2048
Transcript variant c	1, 3a,4	1525
Transcript variant d	1,4	1488

**Table 2:** HFE2 exon positions on chromosome 1

	**Exon 1**	**Exon 2**	**Exon 3a**	**Exon3b**	**Exon 4**
Start	145,413,191	145,414,693	145,415,278	145,415,278	145,416,313
End	145,413,427	145,414,879	145,415,315	145,415,838	145,417,545
Length	236	186	37	560	1232

Lengths are in bps.

From this table, it can be seen that, since exons 3a and 3b overlap each other, the gene prediction process should be able to identify a total of four distinct coding regions in the gene: exon 1, exon 2, exon 3a/3b and exon 4.

The goal of this test is to determine whether each implementation can detect coding regions in the HFE2 gene and to evaluate their processing time. This process is repeated for DNA sequences of different sizes in order to determine how each algorithm performs with varying amounts of data.

For each of these cases, the measured processing time also includes data transfer time. One of the advantages of including data transfer time is that the tests reflect what the user is actually experiencing. In addition, it allows for processing times of larger sequences to be extrapolated linearly. It should be reiterated that these tests are not meant to quantify the reliability of the chosen approach; this type of study has already been well-documented and has shown that frequency analysis is reliable [9,10].

## Availability and requirements

For this paper, an Intel® Core™ i7-2600 K processor (8 MB cache, 3.40 GHz, 8 threads) was used with 8 GB of RAM. The graphics card used was a GeForce GTX 560 1 GB GDDR5 with 336 CUDA cores. It is important to note that, while some may think that the availability of 336 CUDA cores could improve the execution speed by a factor of 336 times compared to a CPU implementation, it is not the case. This is due to the fact that CUDA cores and CPU cores serve different purposes and therefore, are architecturally different. Details concerning CUDA cores can be found in Nvidia’s documentation [24].

### Coding region detection

**Figure 6:**
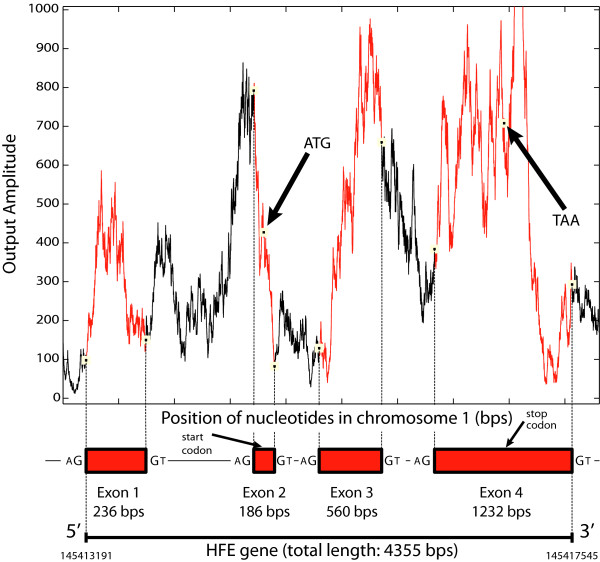
**Frequency component at f**_**s**_**/3 for different areas in the HFE2 gene using a sliding FFT and Goertzel algorithm.**

In the first part of the test, it is important to verify that the coding region detection aspect of each implementation is functional. To do so, it is possible to plot the data of the frequency analyses and compare them with the known structure of the gene [14,25]. MATLAB results show that all implementations proposed in this work produce the same outcome (Figure 6). Each peak in this figure represents a probable coding region and, by comparing the results to the gene structure presented in Tables 1 and 2, it can be shown that the approach is successful at identifying coding regions.

### Processing time

Using the test setup described previously, ten different implementations of the gene prediction algorithm have been evaluated using seven different DNA segment sizes. The results of the test are presented in Table 3.

**Table 3:** Runtime for varying sequence lengths

			**Time (s) for the following sequence sizes:**						
**Function**	**Loop type**	**Processing**	**5,000**	**50,000**	**200,000**	**500,000**	**1,000,000**	**5,000,000**	**15,000,000**
1 goertzel.m	PARFOR	CPU 8 T	1.06	8.29	32.91	82.16	161.89	805.44	TLTC
2 goertzelMEX	FOR	CPU	0.18	1.78	7.11	17.84	35.65	178.30	535.21
3 goertzelMEX	PARFOR	CPU 2 T	0.19	0.99	3.86	9.58	19.20	100.39	287.35
4 goertzelMEX	PARFOR	CPU 4 T	0.18	0.60	2.36	5.81	11.41	56.27	164.84
5 goertzelMEX	PARFOR	CPU 8 T	0.25	0.53	1.95	4.75	9.52	47.49	164.57
**6 Custom Goertzel**	**PARFOR**	**CPU 8 T**	**0.25**	**0.37**	**1.18**	**2.83**	**5.56**	**27.63**	**87.47**
7 JACKET’s FFT (full sequences)	GFOR	GPU	0.03	0.22	0.78	1.90	3.78	18.82	57.68
**8 JACKET’s FFT (1 M blocks)**	**GFOR**	**GPU**	**0.03**	**0.22**	**0.78**	**1.90**	**3.78**	**18.90**	**56.70**
9 Matlab’s FFT	PARFOR	CPU 8 T	0.29	0.42	1.46	3.51	6.95	34.12	109.15
10 Custom Goerztel on GPU	GFOR	GPU	0.22	0.79	2.82	7.15	14.09	71.01	213.31

While the most significant results are shown in that table, many other tests had to be run to ensure that no critical points were missed.

The first implementation considered is the goertzel.m approach where the *goertzel* function was executed. It was found that, when parallelism was not used, the processing time became excessive and required over 4.5 h to process 15 million bps. That option was therefore not considered further. For an implementation using eight threads, running goertzel.m for a sequence of 1 million bps takes 162 s on the test machine. For sequences larger than 5 million bps, it was deemed unnecessary to evaluate the performance as the processing speed was already too low. When the DNA sequences take too much time to process, the table indicates that it is too large to consider (TLTC).

The solution using the GoertzelMEX function was implemented using one, two, four and eight threads. This is done so as to show the performance gained when CPU cores are added. Row 5 of Table 3 shows a nearly 18x improvement over using goertzel.m when processing a 1 million bps sequence with the same eight threads. Rows 3 and 4 show that, even with two or four threads, it is already possible to obtain better results than with goertzel.m using eight threads.

A custom Goertzel algorithm was also tested running on eight threads. Its processing time is presented on the sixth row of Table 3. It shows that, when only the CPU is considered, this approach provides the best results.

The gene prediction algorithm was also implemented with MATLAB’s FFT function and the processing times are presented in Row 9 of Table 3. The results show that the FFT’s processing time is much larger than with the Goertzel implementations. This confirms the fact that the Goertzel algorithm is more efficient than the FFT when the targeted number of frequencies is small.

The two remaining implementations use the FFT function on the GPU. It should be noted that when using the GPU, the FFT is faster than Goertzel and thus the latter is not discussed in greater detail. Nonetheless, results from running Goertzel on the GPU are shown on Row 10 in Table 3. For the first implementation of the FFT, the entire sequence was loaded onto the GPU before processing (Row 7). In the other implementation, the sequence was broken down into smaller blocks of 1 million bps and processed sequentially (Row 8).

It is worth noting that Rows 7 and 8 represent the same operation up to the size of 1 million bps. After that point, the results remain almost identical: sending 15 times a sequence of 1 million bps is only one second faster than sending a large 15 million bps sequence. Our tests have shown that breaking down a large sequence into sizes smaller than 1 million bps results in performance losses whereas sizes larger than 15 million bps yields out of memory errors. According to our results, GPU implementations using the proposed test setup should divide DNA sequences into blocks of 1 to 15 million bps for optimal results.

## Discussion

The tests performed in the previous section show that the choice of algorithm and of implementation plays an important role in the feasibility of gene prediction in MATLAB. Using a straightforward goertzel.m approach with a single thread would not have been possible within a reasonable time frame. Even with eight threads enabled, the extrapolated processing time for 15 million bps would be close to 41 min. When GoertzelMEX and a custom Goertzel algorithm were used, the processing time was reduced to close to 90 s. Finally, with the GPU, the processing time could be reduced to 57 s. These results show that acceleration via a CUDA-enabled graphics card yields the best performance when the sequence to be analyzed is large enough to justify the overhead, yet small enough not to deplete available resources. For a relatively simple yet efficient implementation, a custom Goertzel algorithm using MATLAB’s CPU parallelization (Row 6) can provide results that are about 36% slower than with a GPU (Row 8).

While the absolute difference in processing time may seem inconsequential, it should be noted that frequency analysis is often combined with other techniques to make gene prediction more robust. In addition, to improve on the reliability of the approach, it is sometimes relevant to perform frequency analysis for different window sizes. In those cases, the benefits of using a GPU can be justified. Otherwise, a well-designed MATLAB algorithm running on CPU would provide satisfactory results.

The approach proposed in this paper can deal with a large amount of data in a reasonable time while being accurate and reliable given the proven track record ([9,10]) of the algorithm. It also remains easy to use for researchers who are not experts in the field of bioinformatics. Building upon this foundation, the next step is to use these methods to accurately identify new genes involved in monogenic and complex diseases.

## Conclusions

In this paper, we presented a number of ways of implementing gene prediction using MATLAB. The different implementations were described and evaluated to test for processing time. We have shown that this approach allows for the processing of very large sequences (15 million bps was used) in a reasonable time. This renders the processing of the entire human genome and other organisms very feasible on a conventional desktop machine. It is the authors’ belief that this type of demonstration has never been published before.

In addition, each implementation was also evaluated for shorter DNA sequences to help analyze how the processing time evolves with different sequence lengths. Results show that, with common desktop computers, it is possible to perform gene prediction on sequences of 15 million bps quite rapidly. Using MATLAB’s FFT, an eight-core parallel implementation was able to complete the operation in less than five minutes whereas a GPU-accelerated version did it in approximately one minute. Using a CPU, the work shows that direct access to the MEX function increases execution speed and that the PARFOR construct should be used in order to take full advantage of the parallelizable Goertzel implementation. When the target is a GPU, the work shows that data need to be segmented into manageable sizes within the GFOR construct before processing in order to minimize execution time.

The fact that these results can be achieved within the MATLAB environment without calling upon custom hardware/software solutions means that researchers already familiar with MATLAB can readily use this technique without requiring additional IT resources. The source code provided with this paper can be run locally for accurate results at fast processing speeds ( Additional file 1, Additional file 2). This can help make gene prediction tools more accessible to geneticists and can help speed the discovery of new genes.

## Competing interests

The authors declare that they have no competing interests.

## Authors’ contributions

HTB and RB proposed the initial concept and participated in the design of the study. The detailed study design was developed by the members of the research team, SRR and JGM who also carried out the computations and simulations to produce the published results. All authors have read and approved the final manuscript.

## Authors’ information

Sylvain Robert Rivard received the B.Sc., M.Sc. and Ph. D. degree in molecular genetics from the Université de Montréal, Québec, Canada, in 1984, 1988 and 1995 respectively. He held three Posdoctorals positions (Colorado State University, Fort-Collins, Colorado, USA; Établissement Français du sang, Brest, France and TIGEM Napoli in Italia). He is currently pursuing the M.A.Sc. degree in electrical engineering from Université du Québec à Chicoutimi (UQAC), Canada. His main research interests include human genetics, computational biology and microelectronics.

Jean-Gabriel Mailloux received the B.Eng degree in computer engineering from the Université du Québec à Chicoutimi, Canada, in 2005 where he also received the M.A.Sc. in computer engineering in 2008. He is currently pursuing a Ph. D. in computer engineering. His current research interests include computational biology and optimal approaches for testing algorithms in FPGAs and GPUs.

Rachid Beguenane received his D.E.A. and Ph.D. degrees, both in electrical engineering from CNAM, Paris, France in 1991 and 1994 respectively. During this period, he conducted his Ph.D. research work at École des Mines de Douai, France. From 1995 to 1997, he held a teaching and research position at the Professional Institute of Amiens, France. During 1997/1998, he was a post-doctoral researcher at the School of Information Technology and Engineering of the University of Ottawa, Canada. From 1998 to 2002, he has been employed as ASIC/FPGA design engineer by Telexis inc., Nortel Networks, and Prova Scientific Corp. in Ottawa and Montreal, Canada. He mainly designed and verified IC chips for telecommunication industry. From 2002 to 2009, he was associate professor at Université du Québec à Chicoutimi (UQAC), Canada. Since 2009, he is associate professor in electrical and computer engineering department of Royal Military College of Canada. Dr. Beguenane has authored and co-authored more than 60 technical papers.

Hung Tien Bui (S' 04, M' 06) received the B.Eng. degree in 1998 from Concordia University in electrical engineering in Montreal, Canada and the M.A.Sc. degree in 2000 in computer engineering from Florida Atlantic University. He received the Ph.D. degree in electrical engineering in 2006 from École Polytechnique de Montréal. He has been with the Department of Applied Sciences at Université du Québec à Chicoutimi since 2006 where he is currently an assistant professor. His research interests revolve around high performance microelectronics and signal processing applied to computational biology and health sciences.

## Supplementary Material

Additional file 1 Example code for using JACKET_seq_FFT.m Click here for file

Additional file 2 Frequency analysis on a sequence using JACKET's FFT. Click here for file
